# Wesley LifeForce Suicide Prevention Gatekeeper Training in Australia: 6 Month Follow-Up Evaluation of Full and Half Day Community Programs

**DOI:** 10.3389/fpsyt.2020.614191

**Published:** 2021-01-12

**Authors:** Jacinta Hawgood, Yu Wen Koo, Jerneja Sveticic, Diego De Leo, Kairi Kõlves

**Affiliations:** ^1^Australian Institute for Suicide Research and Prevention, World Health Organisation Collaborating Centre for Research and Training in Suicide Prevention, School of Applied Psychology, Griffith University, Mt Gravatt, QLD, Australia; ^2^Gold Coast Health, Mental Health and Specialist Services, Southport, QLD, Australia

**Keywords:** suicide, suicide prevention, gatekeeper training, Wesley LifeForce, capability, evaluation

## Abstract

**Background and Objective:** Wesley Mission LifeForce training is an Australian suicide prevention gatekeeper program which has not been formally evaluated. The aims of this evaluation were to (1) determine the short- and medium- term impacts of the training on worker capabilities (perceived and declarative knowledge), attitudes, and reluctance to intervene measures; and (2) compare the impact of the half and full day workshops on these measures.

**Method:** 1,079 Australian community workers of diverse professional backgrounds completed a pre-workshop questionnaire as part of registration for the Wesley LifeForce suicide prevention training between 2017 and 2019. Of these, 299 participants also completed the post workshop questionnaires (matched sample). They attended either half day (*n* = 97) or full day workshops (n = 202) and completed also a 3- and 6- month follow-up questionnaire. We used linear mixed-effect modeling for repeated measures to analyze data.

**Results:** LifeForce training participants experienced an increase in perceived capability, declarative knowledge, more positive attitudes and reduced reluctance to intervene, at least in the short term. The program is particularly well targeted for community gatekeepers with no prior training, albeit those with prior training in this study also experienced positive and significant gains on most measured constructs.

**Conclusions:** We found evidence of effectiveness of the Wesley LifeForce training over time, without difference between the short (half day) and longer (full day) formats of delivery. Nevertheless, the latter format offers skills-based and skills rehearsal opportunities, the impacts of which we were unable to measure in this evaluation and should be estimated in the future.

## Introduction

There are several definitions of gatekeeper (GK), a concept that has evolved over time from being simply “a person to whom troubled people are turning for help” (1, p. 39) to those in a position to recognize a crisis and the warning signs that someone may be contemplating suicide (1), or a community member who has some face to face contact with numerous community members as part of their standard role (and who may be trained to identify at risk persons and refer them to appropriate support services) (2). The role of GK can be informally denoted, such as parents, friends, neighbors, sports coach or, formally designated such as teachers, doctors, nurses, police officers, and others who may, as a function of their work role, come into contact with suicidal persons (3).

There is some evidence for gatekeeper training (GKT) as a promising suicide prevention initiative (4), For example, GKT has been found to increase perceived knowledge and declarative knowledge about suicide (5–7); enhance self-efficacy for intervening (8, 9); reduce reluctance to intervene (10, 11); reduce stigma associated with suicide (12) and improve attitudes toward suicide/suicide prevention (13). However, while there is some evidence for the short-term efficacy of GKT, there is less evidence for long-term effects of constructs other than knowledge and self-efficacy (14). Interestingly, there is no evidence for retention of attitudinal change over time (14), which, according to Burnette et al. (15), represents a particularly critical outcome for GTK.

The Wesley LifeForce community suicide prevention training program is part of Wesley Mission's national suicide prevention program, funded by the Commonwealth Department of Health as part of Australia's National Suicide Prevention Strategy. The three main activities of Wesley LifeForce include: a) Suicide Prevention Training, b) Suicide Prevention Networks, and c) Memorial Services. The first of these, Wesley LifeForce Suicide Prevention Training, was the focus of the current evaluation.

GKT programs, such as Wesley LifeForce training, aim at educating volunteers or designated individuals in the community to be able to identify people who may be at-risk of suicide. They are designed specifically to enhance knowledge, attitudes, and skills of the GK in order to enable competency to identify those at risk, determine appropriate action for optimal safety of the person, and make appropriate referrals as necessary (15).

Evaluation of Wesley LifeForce training included Phase 1—review of the appropriateness of the training in terms of alignment with minimum training competencies in content and structure; and Phase 2—evaluation of the short to medium term impacts of the training on GK knowledge, attitudes and skills. Phase 1 evaluation findings can be reviewed in a separate report provided to Wesley Mission (see 2). In brief, the evaluation found that the Wesley LifeForce training complied with nearly all minimum standards and competencies for GKT as defined in the study. Recommendations were made for minor improvement of content-related competencies (associated with key learning outcomes of the program) and more significant modifications to the delivery/structural competencies of the training. All recommendations were subsequently implemented. The current study presents Phase 2 findings of the Wesley LifeForce Suicide Prevention Training Evaluation (an updated edition following implementation of the recommended changes).

The Wesley LifeForce training package was designed to meet the needs of both informal and formal GKs, with the former addressed by community training and the latter via more targeted specialized training (e.g., for aged care nurses and relationship counselors). The aim of the current paper is to evaluate the effects of Wesley LifeForce suicide prevention training program targeted at informal GKs (the general community). Specifically, we aimed to compare and determine impacts of the half day and full day general community training programs on perceptions of capability, declarative knowledge, attitudes toward suicide prevention and reluctance to intervene from before to after training, and at three and six-month follow-up periods.

## Method

### Intervention

The general community training's target audience are persons with moderate to no suicide prevention training and/or those requiring contemporary refresher training. LifeForce community workshops are offered as half day (4 h) or full day (6 h) options, with the latter including more skills-based learning mechanisms using video and role-play activities. The training goals for community training include: Identify people who may be at risk of suicide; Communicate appropriately with a suicidal person; Ask a person if they are considering suicide; Conduct a suicide intervention. Three sessions are covered in the training: Session 1 covers the scope of suicide in Australia (statistics, terminology, definitions, theoretical models); Session 2 examines personal/professional beliefs and attitudes as well as barriers to suicide prevention; and, risk and protective factors, and warning signs and ‘triggers' for suicidality/suicide; and Session 3 bridges understanding to skills-based responses using the S.A.L.T (See, Ask, Listen, and Take the person to help) intervention model to guide knowledge application. This intervention model is unique to Wesley LifeForce training, and therefore any gains in measures of declarative knowledge testing this specific model of intervention is less likely to have been gained from general exposure to suicide prevention education or awareness.

### Study Design and Data Collection

Recruitment of participants to the training was via the Wesley Mission website and related news articles and online community networks' newsletter. More specifically areas of high suicide rate around the country were identified and local organizations were approached to reach local networks. Training is hosted at multiple local community venues within each jurisdiction of Australia (all states and territories), with offerings of community training occurring roughly 4 times per month nationally. Participant numbers at workshops were 10–20 per delivery.

A prospective study design was used, with online questionnaires distributed at four time-points to all community training participants. Registration required completion of the pre-workshop online questionnaire, while responding to the subsequent questionnaires relied on participants' willingness to continue participation in the study, which ran from January 2017 until December 2019. The post-workshop questionnaire was sent soon after the workshop, and the follow-up questionnaires were emailed to attendees at 3- and 6–months after the workshops. Two reminders were sent to participants within 2–3 weeks of each wave of the study. The attrition rates were 72.3% from pre- to post-, 72.9% from post to 3-month follow-up, and 44.4% from 3- to 6-month follow-up. All procedures were approved by the Griffith University Human Research Ethics Committee (2017/241).

Concerning professional background of participants, 30% were medical doctors, 29% were psychologists, 10% were epidemiologists, and 31% were from ‘other' professions (e.g., social worker, student, sociologist, public health professional, teacher, counselor/psychotherapist, analyst, CEO, or pastor).

### Measures

Background information included participants' age, gender, Indigenous status, Culturally and Linguistically Diverse background (CALD), professional role, work status, education, years in suicide prevention role, prior training, and expectation of using training in future.

Outcome measures included reluctance to intervene, perceived capability in suicide prevention, declarative knowledge about LifeForce training learning outcomes, and attitudes toward suicide and suicide prevention. The specific measures were as follows:

*Reluctance to Intervene* is a 9-item scale measuring reluctance to intervene with a suicidal individual (10). Participants rated their level of agreement on a 5-point Likert scale from “strongly disagree” to “strongly agree,” with two items reverse-scored. Each item value is summed for a total score ranging from 9 to 45 where higher values mean less reluctance. This scale had poor internal consistency (α = 0.45) as compared to the original testing results by the authors of the scale (α = 0.68) (10).

*Perceived Capability Scale* is a 15-item scale measuring perceived suicide prevention capabilities on skills and/or knowledge items that may be relevant when acting as a ‘gatekeeper' and assisting someone at risk of suicide, and which are covered in the LifeForce training content (16). Participants are asked to rate their current level of capability on a 5-point Likert scale ranging from “not at all capable” to “highly capable.” A total score ranged from 15 to 75, where higher scores mean higher capability. This scale presented an excellent internal consistency (α = 0.95).

*Declarative Knowledge Scale* was developed to align with the LifeForce learning objectives and outcomes of all training modules (16). It includes 17-items in True/False/Do not know answer format. Correct answers to these questions were ascertained by referring to the workshop training material developed by Wesley Mission. Score equals the percentage of correct answers. This scale showed a good internal consistency (α = 0.73).

Attitudes to Suicide Prevention scale (ASP) is a 14-item self-report scale measuring attitudes toward suicide and suicide prevention (17). Thirteen items use a Likert scale from “strongly agree” to “strongly disagree” and the final item response ranging from “none” to “all.” The responses to these items are scored from one (strongly disagree/none) to five (strongly agree/all) and summed, resulting in a total score ranging from 14 to 70, with higher scores indicating more negative attitudes. This scale had a poor internal consistency (α = 0.47) as compared to the original testing results by the authors of the scale (α = 0.77) (17).

### Statistical Analysis

The outcome measures presented above were used as dependent variables. All scales had a normal distribution (the range for skewness or kurtosis between +1.5 and −1.5). We used linear mixed-effect modeling for repeated measures, which accounts for the correlation between the repeated measures for each individual (18). Moreover, this method also deals with unbalanced data with the assumption that missing data are missing at random and they are not dropped from the analyses.

For the linear mixed-effect regression models, workshop type (full day and half day), time (pre, post, 3- and 6-month follow-up), age group (<35 years; 35+ years), working in suicide prevention (never, 0–12 months, 1–5 years, 5–10 years, 10+ years), gender (male, female, other gender identity), work discipline (community support, health, other), and the workshop type × time interaction, and group were entered as fixed effects. The participant ID variable was included in the random intercept to model for within-person factors at baseline. To reduce multicollinearity, all variables included as fixed effects were centered (19). Time (pre, post, 3-month, and 6-month follow-up) was included as a repeated effect. A First-Order Autoregressive (AR1) and Unstructured (UN) covariance structures were examined using −2 Res Log Likelihood and Akaike's information criterion (AIC). Both structures were applied to the levels of group (workshop group) *person (as workshops were delivered in groups and participants were therefore nested within these groups). Random intercepts for participants were included to model for the correlation of within-person factors at the baseline. The AR1 structure was identified as the model with the best fit with all dependent variables. *Post hoc* analyses for the linear mixed models were conducted with Sidak adjustment. Statistical analysis was conducted in the IBM SPSS 25.0.

## Results

Of the 1,079 participants who completed the pre-workshop questionnaire, 299 (27.7%) participants completed the post-workshop questionnaire and were thus included in the analyses. Of the 299, 81 participants also completed the 3-month and 45 completed the 6-month follow-up survey. There were significant differences between those who completed the post-workshop questionnaire and those who did not by gender (χ2(1) = 0.23, *p* < 0.05), age (χ2(1) = 4.11, *p* < 0.05), and expected training use (χ2(1) = 6.02, *p* < 0.01; Supplementary Table 1).

**Figure 1:**
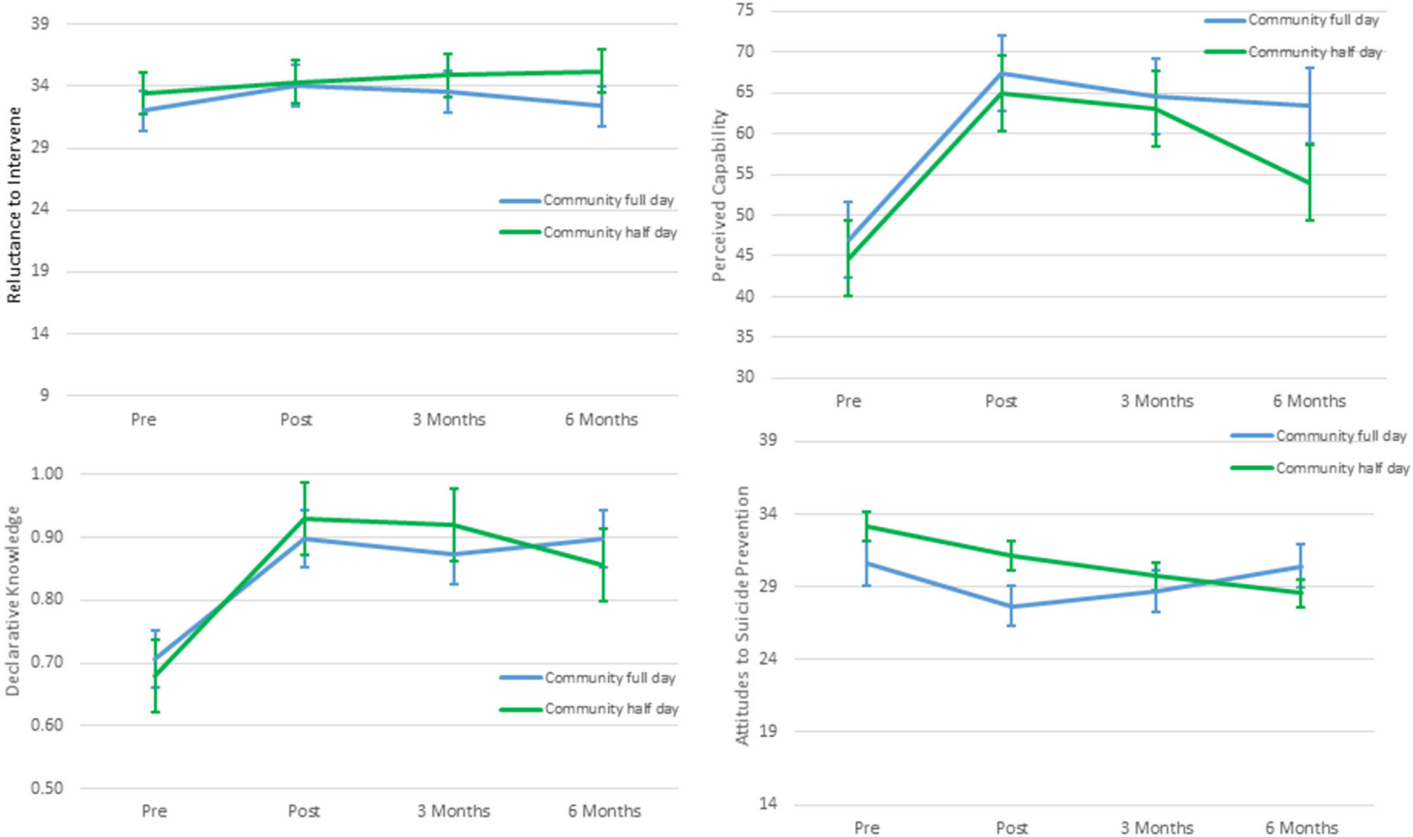
Mean scores with 95% CIs of outcome measures at pre, post, 3-months and 6-month follow-up after community half day and full day Wesley LifeForce training.

A total of 202 participants in the full day and 97 half day workshops were included in the analyses. Demographic information for these participants are presented in Table 1. The only significant differences between the two workshop types are that those who participated in the full day more frequently indicated that they would use the training in the future compared to those in the half-day workshop (χ2(1) = 6.77, *p* < 0.01). Changes in the main outcome measures over the study period are presented in Figure 1.

**Table 1:** Descriptive characteristics of participants included in the study by the workshop type (full day vs. half day training).

		**Community full day (*****N*** **=** **202)**		**Community half day (*****N*** **=** **97)**			
		***N***	**%**	***N***	**%**	***X*^**2**^**	***p***
Gender	Male	30	14.9	14	14.6	0	0.95
	Female	172	85.1	82	85.4		
Age	Below 35 years	37	18.3	25	26.0	2.36	0.13
	35+ years	165	81.7	71	74.0		
Aboriginal and Torres Strait Islander	Aboriginal	9	4.5	5	5.2	0.07	0.79
	Other Australian	193	95.5	92	94.8		
CALD	CALD	23	11.4	11	11.3	0	0.99
	Non-CALD	179	88.6	86	88.7		
State or Territory	ACT	9	4.5	5	5.2		0.78^+^
	NSW	80	39.8	46	47.4	1.64	0.20
	NT	7	3.5	5	5.2		0.53^+^
	QLD	34	16.9	14	14.4	0.28	0.60
	SA	19	9.5	11	11.3	0.27	0.60
	TAS	3	1.5	1	1.0		1.00^+^
	VIC	19	9.5	15	15.5	2.39	0.12
	WA	30	14.9	0	0		<0.001^+^
Work discipline	Community support/carer	93	51.7	43	50.0	0.17	0.92
	Health sector	20	11.1	11	12.8		
	Other	67	37.2	32	37.2		
Working in suicide prevention	Never	63	31.3	41	42.7	4.80	0.31
	0 to 12 months	40	19.9	15	15.6		
	1-5 years	31	15.4	10	10.4		
	5-10 years	32	15.9	12	12.5		
	10+ years	35	17.4	18	18.8		
Previous suicide training		49	25.3	18	19.1	1.32	0.25
Expected to use training		199	98.5	89	92.7	6.77	0.01

*CALD, Culturally and Linguistically Diverse*.

*+Fisher's exact (2-sided) was used for analyses where <80% of the cells have expected values of 5 or less*.

Reluctance to Intervene: Mixed-effects regression analysis (Table 2) showed that time was a significant predictor of the change in mean score of reluctance to intervene (i.e., less reluctance) (*F*_(3, 55.3)_ = 9.74, *p* < 0.001), but not workshop type, nor the interaction of time and workshop type. *Post-hoc* analyses (ST 2) indicated that there was a significant increase in scores from pre-to post-intervention (Mdif = 1.46, 95%CI: 0.71, 2.22; *p* < 0.001), but not from pre to 3-month follow-up (Mdif = 1.49, 95%CI: 0.33, 3.87; *p* = 0.15). There was some decline in scores observed after 3-month follow-up.

**Table 2:** Fixed effect estimates for outcome measures by time, workshop type and workshop type × time interaction^1^.

	**Time**			**Workshop type**			**Workshop type** **×** **Time**		
	***F***	***df^2^***	***p***	***F***	***df^2^***	***P***	***F***	***df^2^***	***p***
Reluctance to intervene	9.74	55.3	<0.001	1.54	149.7	0.22	1.57	55.7	0.21
Perceived capability	258.38	316.7	<0.001	1.71	166.6	0.19	0.58	316.1	0.63
Declarative knowledge	124.81	92.6	<0.001	0.00	151.6	0.95	2.69	92.4	0.05
Attitudes to suicide prevention	16.41	58.3	<0.001	0.46	141.9	0.50	1.70	59.6	0.18

1 *model included also gender, age group, years in suicide prevention, work discipline, training use and prior suicide training (detailed results in ST 3)*.

2 *denominator degrees of freedom. Numerator degrees of freedom are as follows: Time (3), Workshop type (1), Workshop type × Time (3)*.

Perceived Capability Scale: For perceived capability, time was a significant predictor of the change in mean score (*F*_(3, 316.7)_ = 258.38, *p* < 0.001), but not workshop type, nor the interaction. *Post-hoc* analyses (ST 2) indicated that there was a significant increase from pre-to post workshop (Mdif = 20.28, 95%CI: 18.34, 22.22; *p* < 0.001) from pre to 3-month follow-up (Mdif = 17.97, 95%CI: 12.95, 23.75; *p* < 0.001), and from pre to 6-month follow-up (Mdif = 12.76, 95%CI: 5.26, 20.27; *p* < 0.001). Although decline from post to 3-month follow up was not significant, it was significant from 3-month to the 6-month follow-up (Mdif = −7.52, 95%CI: −5.00,−0.03; *p* < 0.05. Younger age (*F*_(2, 127.9)_ = 4.47, *p* < 0.05), longer experience in suicide prevention (*F*_(4, 128.8)_ = 5.36 *p* < 0.001), and previous suicide training (*F*_(1, 129.9)_ = 12.67, *p* < 0.001, ST 3) also predicted higher perceived capability scores across all time periods.

Declarative Knowledge Scale: Time was also a significant predictor of the change in mean score of declarative knowledge (*F*_(3, 92.6)_ = 124.81, *p* < 0.001), but not workshop type. Some time and workshop interaction was observed (*F*_(3, 92.4)_ = 2.69, *p* = 0.051). Nevertheless, *post hoc* analyses revealed that there were no differences between workshop types at any time point. Overall, for both workshops there was a significant increase from pre-to post workshop (Mdif = 0.22, 95%CI: 0.19,0.25; *p* < 0.001, ST 2) from pre to 3-month follow-up (Mdif = 0.20, 95%CI: 0.13,0.27; *p* < 0.001), and from pre to 6-month follow-up (Mdif = 0.18, 95%CI: 0.10,0.26; *p* < 0.001). These gains were maintained with no significant differences from post- to 3-month follow-up, and from 3-month to 6-month follow-up. More experience in suicide prevention (*F*_(4, 121.7)_ = 4.11, *p* < 0.01), and those who had previous suicide training (*F*_(1, 120.2)_ = 5.98, *p* < 0.05) also predicted higher declarative knowledge scores (ST 3) across all time periods.

Attitudes to Suicide Prevention scale (ASP): Similarly, time was a significant predictor of the change in mean score of the attitudes to suicide prevention scale (*F*_(3, 58.3)_ = 16.41, *p* < 0.001), but not workshop type nor the interaction. There were no significant differences at pre-, 3-month, or 6-month follow-up for each type of workshop. However, overall, for both workshops, there was a significant difference from pre-to post workshop (Mdif=-2.46, 95%CI: −3.42,−1.50; *p* < 0.001) and pre to 3-month follow-up (Mdif = −2.66, 95%CI: −5.26,−0.05; *p* = 0.04) indicating a decrease in negative attitudes. Despite these drops (meaning lower negative attitudes) from post- to 6-month follow-up, and from 3-month to 6-month follow-up, these were not significant. Younger age (*F*_(1, 109.9)_ = 4.37, *p* < 0.05), previous suicide training (*F*_(1, 118.5)_ = 4.49, *p* < 0.05) also predicted lower negative attitudes to suicide prevention scores (ST 3).

## Discussion

The main aims of the current study were to evaluate the effects of Wesley LifeForce suicide prevention training targeted at the general community by analyzing the endurance of their impacts on a number of measures, and to compare the impacts of full day and half a day programs. The results support the effectiveness of Wesley LifeForce Suicide Prevention training, for the full and half day training packages for community GKs. All outcome measures including perceptions of capability, declarative knowledge, attitudes toward suicide prevention and reluctance to intervene showed immediate improvements from pre- to post-training. Moreover, these gains were all maintained from post to 3-month, and from 3- and 6-month, with the exception of perceived capability, whereby scores decreased after 3 months follow up.

We did not identify any significant differences in outcomes between participants attending full day or half day workshops. Although there was a significant interaction between workshop types and time for declarative knowledge, *post hoc* analyses indicated there were no significant differences between workshop types at any time. Similarly, Cross et al. (20) compared brief GKT vs. GKT plus behavioral skills training to determine their impacts on skills and use of training and found significant increases for *both* workshops in attitudes and knowledge at post training as well as follow-up (20). However, those who received skills training via role play and behavioral rehearsal showed higher total skills scores (20). It is well established in the GKT literature that knowledge does not necessarily translate to practice (21). A recent systematic review of school-based GKT revealed that only three studies (out of 14) had measured GK behavior/skills changes, which showed generally significant positive effects from pre to post training. However, upon closer examination of these findings, no studies reported maintenance of positive changes and the combined findings implied that the knowledge and skills-based changes may not translate to behavior change (22). However, it was also suggested that this finding may be a result of short follow-up periods during which it is difficult to identify any changes (particularly based on lesser opportunities to apply the skills) (22). Nevertheless, as the application of skills to the real world is the least measured outcome in GKT studies, it is important that such outcomes are included in future investigations.

### Reluctance to Intervene

Related to one's motivation to intervene, this study found less reluctance to intervene with a suicidal person post training, with this difference sustained until the 6-month follow-up. This aligns with other studies that have shown reduced reluctance levels post training, maintained at 5 months post training (10), even when using a randomized control group design (11). However, in the few studies that have looked at the translation of intentions or motivations to intervene following training, there seems to be no association with putting this into practice as measured by self-reported behavioral change (11). As discussed above, while we were unable to measure the behavioral change implications, it would seem important to place additional emphasis within both types of LifeForce training workshops on discussing and practicing ways to overcome potential obstacles to utilization of skills in real-life situations. As stated, only the Lifeforce full day workshop includes skills-based activities, so while we did not find differences between the different formats of delivery, it would be worthwhile measuring specific skills based outcomes between them in the future to better understand their impact of skill utilization. Further, the LifeForce training should pay specific additional emphasis on the role-playing elements of intervention in the context of discussions about the influence of skills-rehearsal on willingness to intervene and reducing discomfort that can often accompany intervention behaviors (23–25). Additionally, some type of professional support or booster training is recommended at least within the 3-months post training to sustain a willingness to respond to and intervene with suicidal persons.

### Perceived Capabilities and Declarative Knowledge

Assessment of perceived capabilities in suicide prevention included examination of a suite of minimum competencies aligned directly with the LifeForce training packages in the form of a self-report measure. This is an important measure as it has been shown previously that confidence in personal abilities can have positive effects on motivating and encouraging participation in suicide prevention activities (15, 26). We found that perceived capability increased post-training and was sustained up to 3-months but decreased at 6-months. This attrition could be related to the lack of opportunity to utilize knowledge and skills over time, despite being unable to report on the opportunities presented to participants to engage suicidal persons during the study period. Nevertheless, it seems fair to assume that informal GKs have much less frequent contact with persons at risk of suicide compared to formally designated GKs whose work necessitates the ongoing use of GK capabilities as part of their role (6).

Our examination of participants' declarative knowledge (a more objective account of assessing suicide prevention facts, directly aligned with LifeForce training learning objectives) showed significantly enhanced knowledge post-training which was maintained over the follow-up period. This is consistent with other findings where GK training has improved suicide-related knowledge in diverse community populations (5–7, 27).

We also found that prior training in suicide prevention and more experience in suicide prevention predicted higher scores on perceived capability and declarative knowledge. Other studies have reported similar associations between prior training and experience with more enhanced training outcomes. For example, GK studies on health professionals (28), and workers from diverse behavioral and health fields (29) have found prior suicide training to be related to greater knowledge and confidence in GKT outcomes. Increased practice and rehearsal of acquired capabilities is known to maintain skills, which may in turn maintain both actual knowledge and perceptions of capabilities (20). Provision of booster training and other supportive education may enhance capability and reinforce acquired skills in the absence of opportunities for intervention. This may be particularly important for informally denoted GKs who are not regularly in contact with suicidal persons.

### Attitudes to Suicide Prevention

Regarding attitudes outcome, we found that negative attitudes to suicide prevention decreased from pre to post-workshop and from pre to 3-month follow-up but not also to 6-month follow-up. Positive attitudinal change toward suicide prevention is one of the most difficult GKT outcomes to sustain long-term, as demonstrated in a recent review by Yonemoto et al. (30) which identified only one RCT study that found attitude changes sustained to 6 months post training among youth helpers (13). We observed that younger age and those with prior training had more positive attitudes, compared to those with no prior training. This demonstrates that regardless of the impacts of LifeForce training, the individual's pre-training experience arguably plays a role in current attitudes toward suicide and suicide prevention. Consistent with the extant literature on attitudes and GKT (15), our results endorse that training generally can result in more positive attitudes for the better, however, this outcome cannot be solely attributed to the impacts of LifeForce workshop.

## Limitations

Our study has several limitations and results should be interpreted against this background. Firstly, in light of the fact that there were some significant differences between completers and non-completers, it is possible that the study suffers from a self-selection bias which may have impacted results. Further, other methodological limitations may prevent causal links being made between LifeForce Training and the enduring participant gains. We did not use a control group to compare different training program effects so were unable to conclude whether competency gains were the result of LifeForce training *per se*, or whether such impacts might be gained from a multitude of other influences. Moreover, not all training attendees participated in the research, and attrition rates were quite high over all time periods; similar experiences were reported by other studies including heterogeneous community samples (11). We attempted to address this limitation through the use of mixed linear modeling as this method accounts for within- and between-participant variance and accounts for correlations between repeated measures for each participant. Finally, scales measuring reluctance to intervene and attitudes to suicide prevention had low internal consistency, in both the original scale development studies and in the current study. Thus, it is possible that results obtained on these scales are not robust enough to be conclusive.

## Conclusion

We found evidence for effective impacts of the Wesley LifeForce training over time, for both the short (half day) and longer (full day) formats of delivery. The latter format offers skills-based and skills rehearsal opportunities which we were unable to measure in this evaluation, but which we recommend be emphasized in future evaluation studies of this program. Specifically, findings revealed that training participants exposed to LifeForce training are likely to experience increased perceived capability, declarative knowledge, positive attitudes and reduced reluctance associated with intervening, at least in the short term. In particular, the program is well targeted for those with no prior training, despite those with prior training also experienced positive and significant gains on nearly all measured constructs. Community members and organizations with different professional background undertaking this training can expect to gain significant learning's and gains in key factors known to impact intervention behaviors.

## Data Availability Statement

We have not provided this statement. The raw data can be made available upon a reasonable request.

## Ethics Statement

The studies involving human participants were reviewed and approved by Griffith University Human Research Ethics Committee. The participants provided consent by progressing past the information sheet informing them that continuation into the online survey will represent their consent to participate.

## Author Contributions

JH designed the study, obtained funding for it, and coordinated the project. YK, JS, and KK analyzed data. JH, YK, and KK wrote the manuscript. All authors critically reviewed the manuscript.

## Conflict of Interest

The authors declare that the research part of the study was funded by the Wesley Mission, however, funding body had no role in study design, data analysis, conclusions, and choice of the journal.

## References

[1] National Strategy for Suicide Prevention Goals and Objectives for Action. Center for Mental Health Services (US). Rockville, MD (2001). 20669520

[2] National Action Alliance for Suicide Prevention (2014) Available online at: https://actionallianceforsuicideprevention.org/sites/actionallianceforsuicideprevention.org/files/Agenda.pdf. [accessed 21 September 2020].

[3] Hawgood J Pasmore K De Leo D Evaluation of Wesley LifeForce Suicide Prevention Training: Phase 1. Brisbane: Australian Institute for Suicide Research and Prevention (2015).

[4] Zalsman G Hawton K Wasserman D van Heeringen K Arensman E Sarchiapone M. Evidence-based national suicide prevention taskforce in Europe: a consensus position paper. Eur Neuropsychopharmacol. (2017) 27:4. 10.1016/j.euroneuro.2017.01.012 28161247

[5] Aseltine Jr RH DeMartino R. An outcome evaluation of the SOS suicide prevention program. Am J Public Health. (2004) 94:446–51. 10.2105/AJPH.94.3.446 14998812 PMC1448274

[6] Arensman E Coffey C Griffin E Van Audenhove C Scheerder G Gusmao R. Effectiveness of depression–suicidal behaviour gatekeeper training among police officers in three European regions: Outcomes of the Optimising Suicide Prevention Programmes and Their Implementation in Europe (OSPI-Europe) study. Int J Soc Psychiatry. (2016) 62:651–60. 10.1177/0020764016668907 27647606

[7] Nasir BF Hides L Kisely S Ranmuthugala G Nicholson GC Black E. The need for a culturally-tailored gatekeeper training intervention program in preventing suicide among Indigenous peoples: a systematic review. BMC Psychiatry. (2016) 16:357. 10.1186/s12888-016-1059-3 27769204 PMC5073837

[8] Clark TR Matthieu MM Ross A Knox KL. Training outcomes from the Samaritans of New York suicide awareness and prevention programme among community-and school-based staff. Br J Soc Work. (2010) 40:2223–38. 10.1093/bjsw/bcq016 28781389 PMC5544031

[9] Chauliac N Brochard N Payet C Duclos A Terra JL. How does gatekeeper training improve suicide prevention for elderly people in nursing homes? A controlled study in 24 Centers. Eur Psychiatry. (2016) 37:56–62. 10.1016/j.eurpsy.2016.05.011 27552322

[10] Wyman PA Brown CH Inman J Cross W Schmeelk-Cone K Guo J. Randomized trial of a gatekeeper program for suicide prevention: 1-year impact on secondary school staff. J Consult Clin Psychol. (2008) 76:104–15. 10.1037/0022-006x.76.1.104 18229988 PMC2771576

[11] Tompkins TL Witt J. The short-term effectiveness of a suicide prevention gatekeeper training program in a college setting with residence life advisers. J Prim Prev. (2009) 30:131–49. 10.1007/s10935-009-0171-2 19283482

[12] Cimini MD Rivero EM Bernier JE Stanley JA Murray AD Anderson DA. Implementing an audience-specific small-group gatekeeper training program to respond to suicide risk among college students: a case study. J Am Coll Health. (2014) 62:92–100. 10.1080/07448481.2013.849709 24456511

[13] Chagnon F Houle J Marcoux I Renaud J. Control-group study of an intervention training program for youth suicide prevention. Suic Life-Threat Behav. (2007) 37:135–44. 10.1521/suli.2007.37.2.135 17521267

[14] Holmes G Clacy A Hermens DF Lagopoulos J. The long-term efficacy of suicide prevention gatekeeper training: a systematic review. Arch Suic Res. (2019) 0:1–31. 10.1080/13811118.2019.1690608 31809659

[15] Burnette C Ramchand R Ayer L. Gatekeeper training for suicide prevention: a theoretical model and review of the empirical literature. RAND Health Q. (2015) 5:16. 28083369 10.7249/rhq-05-01-16 PMC5158249

[16] Hawgood J Sveticic J De Leo D Evaluation of Wesley LifeForce Suicide Prevention Training: Phase 2, Final Report. Brisbane: Australian Institute for Suicide Research and Prevention, Griffith University (2018).

[17] Herron J Ticehurst H Appleby L Perry A Cordingley L. Attitudes toward suicide prevention in front-line health staff. Suic Life-Threat Behav. (2001) 31:342–7. 10.1521/suli.31.3.342.24252 11577918

[18] Detry MA Ma Y. Analyzing repeated measurements using mixed models. JAMA. (2016) 315:407–8. 10.1001/jama.2015.19394 26813213

[19] Kraemer HC Blasey CM. Centring in regression analyses: a strategy to prevent errors in statistical inference. Int J Methods Psychiatric Res. (2004) 13:141–51. 10.1002/mpr.170 15297898 PMC6878533

[20] Cross WF Seaburn D Gibbs D Schmeelk-Cone K White AM Caine ED. Does practice make perfect? A randomized control trial of behavioral rehearsal on suicide prevention gatekeeper skills. J Prim Prev. (2011) 32:195. 10.1007/s10935-011-0250-z 21814869 PMC3249637

[21] Osteen PJ Jacobson JM Sharpe TL Suicide prevention in social work education: how prepared are social work students? J Soc Work Educ. (2014) 50:349–64. 10.1080/10437797.2014.885272

[22] Mo PK Ko TT Xin MQ. School-based gatekeeper training programmes in enhancing gatekeepers' cognitions and behaviours for adolescent suicide prevention: a systematic review. Child Adolesc Psychiatry Mental Health. (2018) 12:29. 10.1186/s13034-018-0233-4 29930701 PMC5992649

[23] Cross W Matthieu MM Lezine DQ Knox KL. Does a brief suicide prevention gatekeeper training program enhance observed skills? Crisis. (2010) 31:149–59. 10.1027/0227-5910/a000014 20573609 PMC2913886

[24] Hendin H Haas AP Maltsberger JT Koestner B Szanto K. Problems in psychotherapy with suicidal patients. Am J Psychiatry. (2006) 163:67–72. 10.1176/appi.ajp.163.1.67 16390891

[25] Barzilay S Yaseen ZS Hawes M Gorman B Altman R Foster A. Emotional responses to suicidal patients: factor structure, construct, and predictive validity of the Therapist Response Questionnaire-Suicide Form. Front Psychiatry. (2018) 9:104. 10.3389/fpsyt.2018.00104 29674979 PMC5895710

[26] Isaac M Elias B Katz LY Belick SL Deane FP Enns MW. Gatekeeper training as a preventative intervention for suicide: a systematic review. Can J Psychiatry. (2009) 54:260–8. 10.1177/070674370905400407 19321032

[27] Bean G Baber KM. Connect: An effective community-based youth suicide prevention program. Suic Life-Threat Behav. (2011) 41:87–97. 10.1111/j.1943-278X.2010.00006.x 21309827

[28] Smith AR Silva C Covington DW Joiner TE Jr. An assessment of suicide-related knowledge and skills among health professionals. Health Psychology. (2014) 33:110. 10.1037/a0031062 23379384

[29] Silva C Smith AR Dodd DR Covington DW Joiner TE. Suicide-related knowledge and confidence among behavioral health care staff in seven states. Psychiatric Serv. (2016) 67:1240–5. 10.1176/appi.ps.201500271 27301763 PMC7871895

[30] Yonemoto N Kawashima Y Endo K Yamada M. Gatekeeper training for suicidal behaviors: a systematic review. J Affect Disord. (2019) 246:506–14. 10.1016/j.jad.2018.12.052 30599375

