# Supplementary material for: Wesley LifeForce Suicide Prevention Gatekeeper Training in Australia: 6 Month Follow-Up Evaluation of Full and Half Day Community Programs
Source: Front Psychiatry. 2021 Jan 12;11:614191. doi: 10.3389/fpsyt.2020.614191 (PMC7835323; doi:10.3389/fpsyt.2020.614191)
Supplement: Supplementary file 1 [file Data_Sheet_1.PDF]

Supplementary Table 1. Descriptive characteristics of non-completers vs completers

		Non completers (*N*=780)		Completers (*N*= 299)			
		N	%	N	%	X^2	*p*
Gender	Male	157	20.2	44	14.8	4.23	.04
	Female	619	79.8	254	85.2		
Age	Below 35years	209	26.8	62	20.8	4.11	.04
	35+ years	571	73.2	236	79.2		
Aboriginal and Torres Strait Islander	Aboriginal and Torres Strait Islander	41	5.3	14	4.7	.15	.70
	Other Australian	739	94.7	285	95.3		
CALD	Yes	114	14.6	34	11.4	1.92	.17
	No	666	85.4	265	88.6		
State and Territory	ACT	39	5.1	14	4.7	7.87	.34
	NSW	262	34.2	126	42.3		
	NT	35	4.6	12	4.0		
	QLD	128	16.7	48	16.1		
	SA	83	10.8	30	10.1		
	TAS	19	2.5	4	1.3		
	VIC	94	12.3	34	11.4		
	WA	105	13.7	30	10.1		
Work discipline	Community support or carer	327	51.9	136	51.1	2.98	.23
	Health sector	97	15.4	31	11.7		
	Other	206	32.7	99	37.2		
Working in suicide prevention	Never	255	33.1	104	35.0	5.88	.21
	0 to 12 months	167	21.7	55	18.5		
	1-5 years	138	17.9	41	13.8		
	5-10 years	88	11.4	44	14.8		
	10+ years	123	16.0	53	17.8		
Previous suicide training		185	24.9	67	23.3	.31	.58
Expected to use training		770	98.8	288	96.6	6.02	.01
Workshop Type	Community full day	506	64.9	202	67.6	.69	.41
	Community half day	274	35.1	97	32.4		

Supplementary Table 2. Linear mixed-effect model post-hoc analyses on outcome measures

	Pre- to Post-Intervention			*Pre- to 3-month follow-up*			*Pre- to 6-month follow-up*		
	Mdif	*p*	*95% CI*	*Mdif*	*p*	*95% CI*	*Mdif*	*p*	*95% CI*
Reluctance to intervene	1.46	<.001	[.71, 2.22]	1.49	.15	[-.29, 3.26]	1.09	.85	[-1.63, 3.81]
Perceived capability	20.28	<.001	[18.34, 22.22]	17.97	<.001	[12.16, 23.79]	12.76	<.001	[5.26, 20.27]
Declarative knowledge	.22	<.001	[.19, .25]	.20	<.001	[.13, .27]	.18	<.001	[.10, .26]
Attitudes to suicide prevention	-2.46	<.001	[-3.42, -1.50]	-2.66	.04	[-5.26, -.05]	-2.39	.64	[-7.03, 2.25]
	Post- to 3-month follow-up			*Post to 6-month follow-up*			*3-month to 6-month follow-up*		
	Mdif	*p*	*95% CI*	*Mdif*	*p*	*95% CI*	*Mdif*	*p*	*95% CI*
Reluctance to intervene	.02	1.00	[-1.80, 1.84]	-.37	1.00	[-3.10, 2.36]	-.39	1.00	[-3.59, 2.80]
Perceived capability	-2.31	.87	[-8.06, 3.45]	-7.52	.05	[-15.00, -.03]	-5.21	.57	[-14.34, 3.92]
Declarative knowledge	-.02	1.00	[-.09, .06]	-.04	.76	[-.12, .04]	-.02	1.00	[-.13, .09]
Attitudes to suicide prevention	-.20	1.00	[-2.80, 2.40]	.07	1.00	[-4.56, 4.70]	.27	1.00	[-4.90, 5.44]

Mdif – Mean difference. A negative and positive mean difference indicates an increase and decrease, respectively

Supplementary Table 3. Fixed effect estimates

	Age group			Gender			Working in suicide prevention			Work discipline			Expected use of training			Previous suicide training		
	F	*df*[1]	*p*	*F*	*df*[1]	*p*	*F*	*df*[1]	*p*	*F*	*df*[1]	*p*	*F*	*df*[1]	*p*	*F*	*df*[1]	*p*
Reluctance to intervene	1.29	114.8	.26	2.36	117.7	.13	.35	119.6	.85	.07	123.9	.94	.17	104.9	.68	.70	124.4	.40
Perceived capability	4.47	127.9	<.05	1.86	130.0	.18	5.36	128.8	<.001	1.15	127.1	.32	2.13	132.7	.15	12.67	129.9	<.001
Declarative knowledge	1.96	119.2	.17	.28	121.2	.60	4.11	121.7	<.01	.50	122.9	.61	.04	125.2	.83	5.98	120.2	<.05
Attitudes to suicide prevention	4.37	109.9	<.05	1.48	115.0	.23	1.84	114.3	.13	1.41	118.8	.25	.07	109.3	.80	4.49	118.5	<.05

Numerator degrees of freedom are as follows: Time (3), Workshop type (1), Workshop type × Time (3), Age Group (2), Sex (1), Years in suicide prevention (4), Work Discipline (2), Previous suicide training (1), Expected training use (1)
